# Differential methylation in rare ophthalmic disorders: a systematic review protocol

**DOI:** 10.1186/s13643-019-0999-3

**Published:** 2019-04-10

**Authors:** Katie Kerr, Helen McAneney, Amy Jayne McKnight

**Affiliations:** Centre for Public Health, Queen’s University Belfast, c/o Regional Genetics Centre, Level A, Tower Block, Belfast City Hospital, Belfast, BT9 7AB Co. Antrim UK

**Keywords:** Association, Epigenetics, Epigenomics, Eye, Inherited, Methylation, Ophthalmic, Rare disease, Systematic review, Vision

## Abstract

**Background:**

Rare ophthalmic conditions often cause degenerative vision loss which leads to loss of independence, ability to work and ultimately quality life. Differential methylation is an epigenomic marker that is a feature of several diseases, including eye conditions. This review will aim to elucidate the extent to which differential methylation has been identified in rare ophthalmic conditions.

**Methods:**

A systematic review will be conducted of articles found in the electronic databases MEDLINE, EMBASE, PubMed and Cochrane Library of Systematic Reviews. Grey literature databases GreyLit and OpenGrey will be searched for relevant unpublished sources. Reference lists of articles which meet eligibility criteria will also be screened for forward and reverse citations. Eligibility criteria will include quantitative articles published, before July 2018, written in English and featuring analysis of differential methylation in rare ophthalmic disorders. Studies will be screened firstly by title, abstract and keywords and then by full text for any remaining sources, following the Preferred Reporting Items for Systematic Reviews and Meta-Analyses (PRISMA) guidelines. Data extraction of key characteristics will be completed using customised forms. Methodological rigour will be assessed using customised forms modelled on the Joanna Briggs Institute critical appraisal forms.

**Discussion:**

This systematic review will enable us to identify if differential methylation can be used to characterise rare ophthalmic disease, which could have crucial implications for improving the accuracy and speed of diagnosis, identifying novel therapeutic targets to reduce or prevent vision loss and overall improving understanding of rare ophthalmic disease.

**Systematic review registration:**

PROSPERO CRD42018094231

**Electronic supplementary material:**

The online version of this article (10.1186/s13643-019-0999-3) contains supplementary material, which is available to authorized users.

## Background

Rare diseases are defined by the European Union as those which affect less than 5 in 1000 people; yet, cumulatively 30 million people in the UK are affected by a rare disease, or 1 in 17 [1]. There are over 8000 different type of rare diseases, many of which have ophthalmic impact, and phenotypic heterogeneity means that two in five patients struggle to obtain a diagnosis [2]. Even where a diagnosis can be obtained, treatment options are often limited [3]. In the case of common complex ophthalmic conditions, such as diabetic retinopathy, early detection and management can prevent blindness [4]. Unfortunately, the lack of diagnosis and treatment in several rare ophthalmic conditions results in degeneration of patient vision. Therefore, several research teams worldwide are attempting to improve understanding of rare ophthalmic conditions, with emphasis on improving diagnosis and therapeutic development through genetic analysis, including the UK based 100,000 Genome’s Project [5], the US-based National Ophthalmic Disease Genotyping and Phenotyping Network (eyeGENE, [6]) and the Irish Target 5000—Gateway to Vision [7]. This research has provided a platform for epigenetic analyses, such as the study of differential methylation between rare ophthalmic diseases, which may further facilitate diagnosis and therapeutic research.

Methylation is a reversible post-translational modification to DNA. [8] Changes in methylation can occur for short or long periods because of heritable, somatic or environmental factors, with the potential to persist over multiple generations. Differential methylation is a key epigenomic biomarker that has been observed in several diseases including several cancers [9], neurological conditions such as Alzheimer’s disease and Autism Spectrum Disorder [10], rare renal disorders (Kerr et al., submitted) and several common eye conditions such as age-related macular degeneration [11] and cataracts [12]. Therefore, differential methylation may play an important role in characterising rare ophthalmic conditions.

To date, no reviews into differential methylation and rare ophthalmic disease have been conducted. However, several recent studies emphasise the important role methylation may have in ophthalmic development and disease [13–15]. Therefore, there is a need to summarise and critically appraise available evidence, to inform the global debate on what is already known and to evaluate where gaps still exist.

### Review aim and objectives

#### Aim

To determine what evidence currently exists of differential methylation in rare eye diseases.

#### Objectives

- Systematically search for current literature relevant to methylation and rare ocular diseases
- Synthesise data to determine the method of methylation measurement and methodological rigour of any such studies
- Discuss differential methylation as a biomarker of rare eye disease

## Methods

This protocol is registered in PROSPERO (ID CRD42018094231) and will follow the Preferred Reporting Items for Systematic Review and Meta-Analysis Protocols (PRISMA-P) guidelines (Additional file 1).

### Inclusion criteria

Eligibility criteria for studies to be included in the review were designed using the Population, Intervention, Comparison, Outcome (PICO) framework [16], (with methylation seen as an exposure rather an intervention). To conduct a wide search encapsulating as many studies as possible, all quantitative study designs that meet inclusion criteria will be considered. The population will include patients, animal models, and in vitro models of rare ophthalmic diseases. However, as DNA methylation patterns will vary between these models, a separate quantitative analysis of the different models will be conducted if studies in these different environments are returned in the search. Conditions with ophthalmic impact, but which are not primarily ophthalmic diseases, are not eligible for inclusion (such as Behҫet’s syndrome). Studies can include both male and female participants of all ages and ethnicities. The focus of articles considered for this review will be the analysis of observed methylation levels in rare ophthalmic disease(s) compared to common ophthalmic disease(s) and/or normal controls. For this review, the comparator element will not be applicable. Outcomes reviewed will be identification of differential methylation and its potential for its analysis to improve understanding of rare ophthalmic conditions, improving accuracy or speed of diagnosis, identification of novel therapeutic targets, or any way in which differential methylation analysis could be applied to improve patient quality of life. Only articles written in English will be eligible for inclusion.

### Search strategy

The electronic databases MEDLINE, EMBASE, PubMed and Cochrane Library of Systematic Reviews will be searched for articles published before July 2018. This will be followed by a search for unpublished articles, or grey literature, using the databases GreyLit and OpenGrey. Additionally, we will screen reference lists of included studies for potentially eligible studies, as well as articles which have cited the source to identify any forward/reverse citations.

Search terms will be created initially for MEDLINE via Ovid, subsequently adapted for other databases, and will encompass different terms for methylation and many rare ophthalmic conditions (see Additional file 2: Appendix 1, an example search). As no registry of all rare ophthalmic conditions exists, disease-based search terms will be derived by identifying conditions from a number of sources including the ophthalmic inclusion criteria from the 100,000 Genomes Project [17] National Eye Institute [18], the Genetic and Rare Disease Information Center [19] and Retina International [20].

### Study selection

The screening and data extraction process will be carried out independently by two personnel, by following the checklist for eligibility characteristics (type of study population, intervention, comparison and study design), initially screening the titles and abstracts of identified studies to assess their eligibility for inclusion, then followed by the screening of full texts to make the final decision regarding the inclusion of each study. Any discrepancies will be discussed; with the option to bring in a third party should no agreement be reached. A PRISMA study selection flow diagram [21] of this process will be produced.

Data will be managed using reference management software, (Endnote X8, Clarivate Analytics), including storing articles and reasons for exclusions. Articles which do not meet the language eligibility criteria will first be removed. Titles, abstracts and keywords will then be screened to remove duplicates and articles overtly not relevant to differential methylation in rare ophthalmic conditions. Additionally, reference lists of any relevant systematic review articles identified will be screened to ensure no primary research studies are missed. Finally, articles will then be screened by reading the full text. The remaining articles which meet the criteria for inclusion in this review will then undergo data collection and critical appraisal.

### Data extraction

Data will be extracted into customised forms. Variables extracted will include:

I. Study characteristics such as country of origin, year of publication and sample size
II. Design information such as case-control
III. Participant information such as ages, gender, ethnicities, medical history
IV. Experimental information such as methylation measurement method
V. Key outcomes such as identification of differential methylation as a prognostic biomarker of rare ophthalmic disease

Where necessary, we will contact the authors of included studies for additional information.

The proposed study is a secondary analysis of peer-reviewed publications. No human subjects will be directly involved. Therefore, this study does not require approval from an ethical research committee.

### Critical appraisal of studies

Critical appraisal of study methodological rigour will be performed using customised forms based on the Joanna Brigg’s critical appraisal tools [22], depending on the experimental design of the study being assessed (see Additional file 2: Appendix 2 and 3 for examples of case-control and case report critical appraisal forms). Studies will be allocated weak, moderate or strong methodological rigour based on sample size, appropriate matching of cases to controls, method of methylation measurement, valid statistical analysis, use of experimental controls and attempts to deal with confounding factors.

### Data synthesis

If sufficient data is available, that is, that there are three or more studies assessing the same intervention and provide adequate data on the same outcomes, a meta-analysis will be performed. Review Manager (version 5.3) will be used to perform a fixed or random effect model meta-analysis, dependent on the homogeneous characteristics of the included studies. To detect statistical heterogeneity, we will consider the *I*^2^ statistic and *χ*^2^ tests across the included studies, where *I*^2^ < 25% will be considered as low, 25–50% moderate and 50 + % as high degrees of heterogeneity. A sensitivity analysis of the estimates of the effects will be carried out by deleting each study in turn, in case a statistically significant result depends on a single study. Should a moderate degree of heterogeneity of studies exist, we will assess the studies thoroughly to determine the potential source of heterogeneity and conduct further subgroup analysis if necessary. For example, subgroup analysis may be appropriate between different rare ophthalmic disorders, or between participants of varying age, sex and those with co-morbidities. Furthermore, publication bias will be assessed by visual analysis of funnel plots for evidence of asymmetry followed by Eggers regression test. If there are marked methodological variations, or high levels of heterogeneity, amongst the included studies, we will not perform a meta-analysis. If a meta-analysis cannot be performed, or if insufficient data exists, a narrative synthesis will be undertaken. Such a narrative synthesis would be performed using the University of Lancaster narrative synthesis guidelines. [23]

## Discussion

Differential methylation has been identified in several common complex diseases including but not limited to differential methylation observed in age-related macular degeneration [5], epigenetic suppression in age-related cataracts [24] and changes in DNA methylation as a result of hypoxia in glaucoma [25]. This systematic review will evaluate the evidence for differential methylation in rare ophthalmic disease compared to normal controls and common ophthalmic conditions, as well as the implications for improving our understanding of the rare ophthalmic disease. Outcomes of particular interest will be any discussion of utilising differential methylation as a diagnostic or prognostic biomarker, or additionally identifying any novel therapeutic target. This will be relevant to both clinicians and researchers working with rare ophthalmic disease patients. Whilst we anticipate that this review will uncover mostly observational design studies (due to the scarce nature of individual rare diseases and previous studies of differential methylation in other diseases of mostly observational design), this review is a crucial step towards developing a panel of clinically useful biomarkers which can be used to aid patients with rare ophthalmic diseases where accurate diagnosis and effective treatment is currently limited.

## Additional files

Additional file 1: PRISMA-P 2015 Checklist. (DOCX 32 kb)

Additional file 2: Appendix 1. Search terms used in MEDLINE and adapted for other databases. Appendix 2. Template quality appraisal form for case control studies. Appendix 3. Template quality appraisal form for case report studies. (DOCX 30 kb)
