# Supplementary material for: Differential methylation in rare ophthalmic disorders: a systematic review protocol
Source: Syst Rev. 2019 Apr 10;8:93. doi: 10.1186/s13643-019-0999-3 (PMC6456967; doi:10.1186/s13643-019-0999-3)
Supplement: Supplementary file 2 — Appendix 1. Search terms used in MEDLINE and adapted for other databases. Appendix 2. Template quality appraisal form for case control studies. Appendix 3. Template quality appraisal form for case report studies. (DOCX 30 kb) [file 13643_2019_999_MOESM2_ESM.docx]

**Differential methylation in rare ophthalmic disorders: a systematic review protocol.**

Katie Kerr, Dr Helen McAneney, Dr Amy Jayne McKnight

**Appendix**

| **Appendix 1.** Search terms used in MEDLINE and adapted for other databases. | |
| --- | --- |
| **#** | **Search term** |
|  | (Achromatopsia or ACHM or Pingelapese blindness or Rod monochromacy or Rod monochromatism).mp. |
|  | (Acute zonal occult outer retinopathy or AZOOR).mp. |
|  | (Adult-onset vitelliform macular dystrophy or Adult-onset foveomacular dystrophy or Gass disease or Pseudo-Best disease or Pseudo-vitelliform macular dystrophy).mp. |
|  | (Aland islands eye disease or Forsius-Eriksson syndrome or Forsius-Eriksson type ocular albinism).mp. |
|  | (Anterior uveitis or Iridocyclitis).mp. |
|  | (Autosomal dominant keratitis or Hereditary Keratitis).mp. |
|  | (Autosomal recessive isolated optic atrophy or Autosomal recessive nonsyndromic optic atrophy).mp. |
|  | (Autosomal recessive primary microcephaly or Microcephalia vera).mp. |
|  | (Axenfeld-Rieger syndrome or Axenfeld syndrome or Rieger syndrome).mp. |
|  | (Best Disease or Best vitelliform macular dystrophy or Early-onset vitelliform macular dystrophy or Juvenile-onset vitelliform macular dystrophy or Polymorphic vitelline macular degeneration or Vitelliform macular dystrophy type 2).mp. |
|  | (Biettis Crystalline Dystrophy or Bietti crystalline corneoretinal dystrophy).mp. |
|  | (Bilateral corneal abnormality or bilateral corneal abnormalities).mp. |
|  | (Birdshot chorioretinopathy or Birdshot chorioretinitis or Birdshot retinochoroiditis or Birdshot retinochoroidopathy or Vitiliginous choroiditis).mp. |
|  | (Bradyopsia or Prolonged electroretinal response suppression).mp. |
|  | (Central areolar choroidal dystrophy or Central areolar choroidal sclerosis).mp. |
|  | (Choroideremia or Tapetochoroidal dystrophy).mp. |
|  | (Coats disease or Congenital retinal telangiectasia or Leber miliary aneurysm).mp. |
|  | (Congenital cataract or Cataract Hutterite type or Early-onset partial cataract or Total early-onset cataract or Cerulean cataract or Blue-dot cataract or Cataract microcornea syndrome).mp. |
|  | (Congenital foveal retinoschisis or foveal hypoplasia).mp. |
|  | (Congenital glaucoma or Juvenile glaucoma or Hereditary glaucoma or Primary glaucoma).mp. |
|  | (Congenital macula abnormality or North Carolina Macular dystrophy).mp. |
|  | (Congenital microcoria or Congenital miosis).mp. |
|  | (Doyne honeycomb retinal dystrophy or Familial drusen).mp. |
|  | (Duane syndrome or Stilling-Turk-Duane syndrome or Duane retraction syndrome).mp. |
|  | (Eales disease or Idiopathic retinal perivasculitis or Idiopathic retinal vasculitis).mp. |
|  | (Fish-eye disease or Partial LCAT deficiency).mp. |
|  | (Goldmann-Favre syndrome or Enhanced S-cone syndrome).mp. |
|  | (Gyrate atrophy or Hyperornithinemia or Ornithine aminotransferase deficiency).mp. |
|  | (Idiopathic retinal vasculitis-aneurysms-neuroretinitis syndrome or IRVAN syndrome).mp. |
|  | (Isolated anophthalmia-microphthalmia syndrome or Microphthalmia-anophthalmia-coloboma spectrum or MAC spectrum or Colobomatous microphthalmia or Nanophthalmia).mp. |
|  | (Isolated congenital megalocornea or Congenital anterior megalophthalmia).mp. |
|  | (Isolated optic neuritis or Chronic relapsing inflammatory optic neuropathy).mp. |
|  | (Leber Congenital Amaurosis or Early-Onset Severe Retinal Dystrophy).mp. |
|  | (Microcornea-rod-cone dystrophy-cataract-posterior staphyloma syndrome or MRCS syndrome).mp. |
|  | (Norrie disease or Atrophia bulborum hereditaria or Episkopi blindness or Norrie-Warburg disease).mp. |
|  | (Oguchi disease or Oguchi syndrome or Congenital stationary night blindness or Oguchi type or Rod dysfunction syndrome or fundus albipunctatus).mp. |
|  | (Oligocone trichromacy or Oligocone syndrome).mp. |
|  | (Stern-Lubinsky-Durrie syndrome or Corneodermatoosseous syndrome).mp. |
|  | (Superior limbic keratoconjunctivitis or Theodore's superior limbic keratoconjunctivitis).mp. |
|  | (Tolosa Hunt syndrome or Painful ophthalmoplegia).mp. |
|  | (Usher Syndromes or Retinitis pigmentosa-deafness syndrome).mp. |
|  | (Vernal keratoconjunctivitis or Spring catarrh).mp. |
|  | “Retinopathy of prematurity”/ |
|  | Albinism, Ocular/ |
|  | ANIRIDIA/ |
|  | Autosomal dominant optic atrophy.mp. |
|  | Borholm eye disease.mp. |
|  | Brittle cornea syndrome.mp. |
|  | Chandler's syndrome.mp. |
|  | COLOBOMA/ |
|  | Cone Rod Dystrophies/ |
|  | Congenital ectropion.mp. |
|  | Congenital extraocular muscle fibrosis.mp. |
|  | Congenital primary aphakia.mp. |
|  | Corneal Dystrophies, Hereditary/ |
|  | Familial conjunctiva pterygium .mp. |
|  | Familial exudative vitreoretinopathy.mp. |
|  | Fuchs' Endothelial Dystrophy/ |
|  | Fuchs heterochromic iridocyclitis.mp. |
|  | Hereditary vascular retinopathy.mp. |
|  | Infantile nystagmus.mp. |
|  | Inherited macular dystrophy.mp. |
|  | Iridocorneal endothelial syndrome.mp. |
|  | Isolated ectopia lentis.mp. |
|  | Juvenile Macular Degeneration.mp. |
|  | Kearns-Sayre Syndrome/ |
|  | Keratoconus/ |
|  | Late-onset retinal degeneration.mp. |
|  | Lenz microphthalmia syndrome.mp. |
|  | Morning glory syndrome.mp. |
|  | Oculoauriculofrontonasal syndrome.mp. |
|  | Oculocerebral dysplasia.mp. |
|  | Oculomotor apraxia Cogan type.mp. |
|  | Optic perineuritis.mp. |
|  | PANUVEITIS/ |
|  | Patterned dystrophy retinal pigment epithelium.mp. |
|  | Peters anomaly.mp. |
|  | Progressive bifocal chorioretinal atrophy.mp. |
|  | Retinitis Pigmentosa/ |
|  | Ring dermoid of cornea.mp. |
|  | Snowflake vitreoretinal degeneration.mp. |
|  | Stargardt Disease.mp. |
|  | Syndromic microphthalmia.mp. |
|  | Visual snow syndrome.mp. |
|  | Vitreoretinochoroidopathy dominant.mp. |
|  | X-linked retinoschisis.mp. |
|  | (rare eye disease* or rare eye disorder* or rare ophthalmic disease* or rare ophthalmic disorder*).mp. |
|  | (methylation or methylated or hypermethylated or hypomethylated or hypermethylation or hypomethylation).mp. |
|  | 1 or 2 or 3 or 4 or 5 or 6 or 7 or 8 or 9 or 10 or 11 or 12 or 13 or 14 or 15 or 16 or 17 or 18 or 19 or 20 or 21 or 22 or 23 or 24 or 25 or 26 or 27 or 28 or 29 or 30 or 31 or 32 or 33 or 34 or 35 or 36 or 37 or 38 or 39 or 40 or 41 or 42 or 43 or 44 or 45 or 46 or 47 or 48 or 49 or 50 or 51 or 52 or 53 or 54 or 55 or 56 or 57 or 58 or 59 or 60 or 61 or 62 or 63 or 64 or 65 or 66 or 67 or 68 or 69 or 70 or 71 or 72 or 73 or 74 or 75 or 76 or 77 or 78 or 79 or 80 or 81 or 82 or 83 or 84 or 85 or 86 or 87 |
|  | 88 and 89 |

**Appendix 2.** Template quality appraisal form for case control studies.

|  |  | Yes | No | Unclear | Not Applicable | Comments |
| --- | --- | --- | --- | --- | --- | --- |
| 1 | Was the primary focus of the paper methylation? |  |  |  |  |  |
| 2 | Were the groups comparable other than the presence of disease in cases or the absence of disease in controls? |  |  |  |  |  |
| 3 | Were cases and controls matched appropriately? |  |  |  |  |  |
| 4 | Was methylation measured in in a standard reliable way? |  |  |  |  |  |
| 5 | Was methylation measured in the same way for cases and controls? |  |  |  |  |  |
| 6 | Were confounding factors identified? |  |  |  |  |  |
| 7 | Were strategies to deal with confounding factors identified? |  |  |  |  |  |
| 8 | Was the exposure period of interest long enough to be meaningful? |  |  |  |  |  |
| 9 | What statistical analysis was used and was this analysis appropriate? |  |  |  |  |  |
| 10 | Were experimental controls used? |  |  |  |  |  |

**Appendix 3.** Template quality appraisal form for case report studies

|  | | Yes | No | Unclear | Not Applicable | Comments |
| --- | --- | --- | --- | --- | --- | --- |
| 1 | Was the primary focus of the paper methylation? |  |  |  |  |  |
| 2 | Was the patient’s history described clearly? |  |  |  |  |  |
| 3 | Was the patient's current clinical condition described clearly? |  |  |  |  |  |
| 4 | Was methylation measured in in a standard reliable way? |  |  |  |  |  |
| 5 | Were confounding factors identified? |  |  |  |  |  |
| 6 | Were strategies to deal with confounding factors identified? |  |  |  |  |  |
| 7 | Were experimental controls used? |  |  |  |  |  |
